# Combinação de Ferramentas de Telecardiologia para Estratificação de Risco Cardiovascular na Atenção Primária: Dados do Estudo PROVAR+

**DOI:** 10.36660/abc.20230653

**Published:** 2024-03-21

**Authors:** Lucas Leal Fraga, Bruno Ramos Nascimento, Beatriz Costa Haiashi, Alexandre Melo Ferreira, Mauro Henrique Agapito Silva, Isabely Karoline da Silva Ribeiro, Gabriela Aparecida Silva, Wanessa Campos Vinhal, Mariela Mata Coimbra, Cássia Aparecida Silva, Cristiana Rosa Lima Machado, Magda C. Pires, Marina Gomes Diniz, Luiza Pereira Afonso Santos, Arthur Maia Amaral, Lucas Chaves Diamante, Henrique Leão Fava, Craig Sable, Maria Carmo Pereira Nunes, Antonio Luiz P. Ribeiro, Clareci Silva Cardoso

**Affiliations:** 1 Universidade Federal de Minas Gerais Hospital das Clínicas Belo Horizonte MG Brasil Hospital das Clínicas da Universidade Federal de Minas Gerais – Serviço de Cardiologia e Cirurgia Carvdiovascular, Belo Horizonte, MG – Brasil; 2 Hospital Madre Teresa Serviço de Hemodinâmica Belo Horizonte MG Brasil Hospital Madre Teresa – Serviço de Hemodinâmica, Belo Horizonte, MG – Brasil; 3 Universidade Federal de Minas Gerais Faculdade de Medicina Departamento de Clínica Médica Belo Horizonte MG Brasil Universidade Federal de Minas Gerais – Departamento de Clínica Médica – Faculdade de Medicina, Belo Horizonte, MG – Brasil; 4 Universidade Federal de Minas Gerais Hospital das Clínicas Centro de Telessaúde Belo Horizonte MG Brasil Hospital das Clínicas da Universidade Federal de Minas Gerais – Centro de Telessaúde, Belo Horizonte, MG – Brasil; 5 Universidade Federal de São João del Rei Campus Centro-Oeste Dona Lindu Divinópolis MG Brasil Universidade Federal de São João del Rei – Campus Centro-Oeste Dona Lindu – Campus Divinópolis, Divinópolis, MG – Brasil; 6 Universidade Federal de Minas Gerais Instituto de Ciências Exatas Departamento de Estatística Belo Horizonte MG Brasil Universidade Federal de Minas Gerais – Instituto de Ciências Exatas – Departamento de Estatística, Belo Horizonte, MG – Brasil; 7 Faculdade de Ciências Médicas de Minas Gerais Faculdade de Medicina Belo Horizonte MG Brasil Faculdade de Ciências Médicas de Minas Gerais – Faculdade de Medicina, Belo Horizonte, MG – Brasil; 8 Universidade Federal de Ouro Preto Departamento de Medicina Ouro Preto MG Brasil Universidade Federal de Ouro Preto – Departamento de Medicina, Ouro Preto, MG – Brasil; 9 Children's National Health System Washington District of Columbia EUA Children's National Health System – Cardiology, Washington, District of Columbia – EUA

**Keywords:** Doenças Cardiovasculares, Programas de Rastreamento, Telemedicina, Eletrocardiografia, Grau de Risco

## Abstract

**Fundamento:**

As ferramentas de telecardiologia são estratégias valiosas para melhorar a estratificação de risco.

**Objetivo:**

Objetivamos avaliar a acurácia da tele-eletrocardiografia (ECG) para predizer anormalidades no ecocardiograma de rastreamento na atenção primária.

**Métodos:**

Em 17 meses, 6 profissionais de saúde em 16 unidades de atenção primária foram treinados em protocolos simplificados de ecocardiografia portátil. Tele-ECGs foram registrados para diagnóstico final por um cardiologista. Pacientes consentidos com anormalidades maiores no ECG pelo código de Minnesota e uma amostra 1:5 de indivíduos normais foram submetidos a um questionário clínico e ecocardiograma de rastreamento interpretado remotamente. A doença cardíaca grave foi definida como doença valvular moderada/grave, disfunção/hipertrofia ventricular, derrame pericárdico ou anormalidade da motilidade. A associação entre alterações maiores do ECG e anormalidades ecocardiográficas foi avaliada por regressão logística da seguinte forma: 1) modelo não ajustado; 2) modelo 1 ajustado por idade/sexo; 3) modelo 2 mais fatores de risco (hipertensão/diabetes); 4) modelo 3 mais história de doença cardiovascular (Chagas/cardiopatia reumática/cardiopatia isquêmica/AVC/insuficiência cardíaca). Foram considerados significativos valores de p < 0,05.

**Resultados:**

No total, 1.411 pacientes realizaram ecocardiograma, sendo 1.149 (81%) com anormalidades maiores no ECG. A idade mediana foi de 67 anos (intervalo interquartil de 60 a 74) e 51,4% eram do sexo masculino. As anormalidades maiores no ECG se associaram a uma chance 2,4 vezes maior de doença cardíaca grave no ecocardiograma de rastreamento na análise bivariada (OR = 2,42 [IC 95% 1,76 a 3,39]) e permaneceram significativas (p < 0,001) após ajustes no modelo 2 (OR = 2,57 [IC 95% 1,84 a 3,65]), modelo 3 (OR = 2,52 [IC 95% 1,80 a 3,58]) e modelo 4 (OR = 2,23 [IC 95% 1,59 a 3,19]). Idade, sexo masculino, insuficiência cardíaca e doença cardíaca isquêmica também foram preditores independentes de doença cardíaca grave no ecocardiograma.

**Conclusões:**

As anormalidades do tele-ECG aumentaram a probabilidade de doença cardíaca grave no ecocardiograma de rastreamento, mesmo após ajustes para variáveis demográficas e clínicas.

**Figure:**
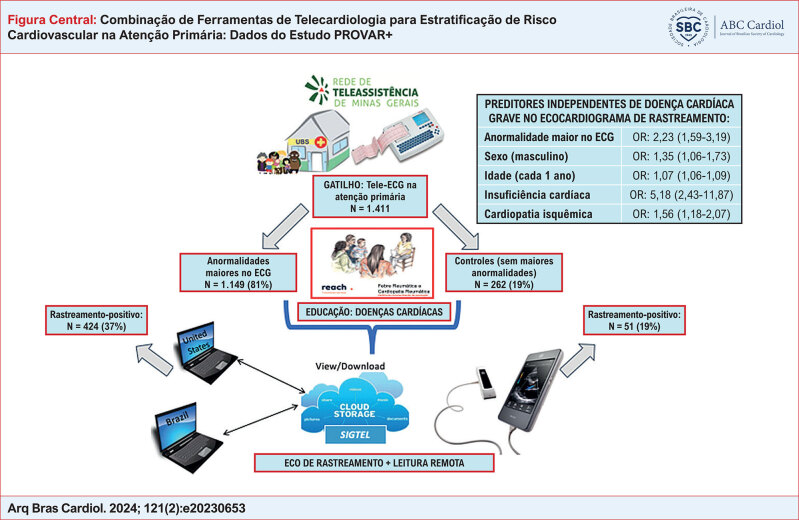
ECG: eletrocardiografia; ECO: ecocardiograma.

## Introdução

O Brasil enfrenta uma alta incidência de doenças cardiovasculares como a causa principal de mortalidade, padrão semelhante ao observado em países desenvolvidos.^1^ Porém, por ser um país em desenvolvimento com um sistema de saúde único e universal (Sistema Único de Saúde – SUS), surgem desafios significativos na busca de um equilíbrio entre a prevenção e o tratamento, em um cenário epidemiológico que mescla características de países desenvolvidos e em desenvolvimento.^2^ Portanto, é essencial adotar estratégias eficazes para melhorar a alocação de recursos de saúde, com foco na prevenção. A realocação de recursos para áreas prioritárias requer uma abordagem estratégica que exige tecnologia e conhecimento médico.

Uma ferramenta crucial para o rastreamento cardiovascular é o ecocardiograma, que permite detectar, classificar e estratificar o risco de doença cardíaca (DC) estrutural. Graças ao desenvolvimento da tecnologia, a ecocardiografia tem se tornado progressivamente portátil e acessível, passando dos centros de imagem para os locais de atendimento, com potencial para chegar a ambientes não atendidos.^3 , 4^ Portanto, uma abordagem em duas etapas, que inclui a realização do ecocardiograma por não especialistas, seguida de interpretação remota por cardiologistas credenciados, é uma estratégia de rastreamento promissora.^5 , 6^ Isso pode permitir uma alocação de recursos mais eficaz e a implementação de programas de rastreamento mais amplos, permitindo um diagnóstico mais precoce e um tratamento imediato, especialmente onde os recursos são escassos.

Atualmente, o SUS permite que médicos da atenção primária (AP) solicitem ecocardiografia para qualquer finalidade diagnóstica, e os pacientes são priorizados apenas com base na data da solicitação. As longas filas e a falta de um sistema de priorização para casos mais urgentes frequentemente levam a atrasos no diagnóstico, contribuindo para resultados desfavoráveis.^5 , 6^ Neste cenário, uma vez que os sistemas de saúde locais têm uma disponibilidade extremamente limitada de exames especializados e encaminhamentos para atenção secundária, longos tempos de espera são comumente observados. Além disso, são frequentes os encaminhamentos de pacientes sem alterações clínicas significativas para exames especializados, contribuindo para longos atrasos.^7^ Na tentativa de solucionar esse problema, foi demonstrado que a adição do ecocardiograma de rastreamento a um escore de estratificação de risco clínico pode ser uma ferramenta promissora para priorizar encaminhamentos para consultas convencionais de ecocardiografia e cardiologia, o que pode potencialmente resultar na redução das listas de espera em áreas sub-atendidas e em melhor alocação de recursos de saúde.^5 , 7^

Uma importante ferramenta para expansão da ecocardiografia portátil é a telemedicina, pois permite a pronta interpretação remota por especialistas, especialmente em regiões de maior vulnerabilidade social.^5 – 7^ A Rede de Telessaúde de Minas Gerais foi implementada em 2005 pelo Governo do Estado de Minas Gerais, com o objetivo de conectar hospitais universitários aos serviços de saúde locais, oferecendo suporte aos profissionais de saúde por meio de tele-eletrocardiografia (ECG), teleassistência e, mais recentemente, tele-ecocardiografia, além de fornecer suporte por meio de teleconsultas.^8^ O sistema de tele-ECG já está disponível em todo o país, com recente expansão para outras nações da América do Sul.^9^ No presente estudo, objetivamos avaliar a acurácia da tele-ECG em locais remotos para predizer anormalidades no ecocardiograma de rastreamento na AP brasileira, como uma estratégia combinada para estratificação de risco.

## Métodos

Os procedimentos e métodos do presente estudo serão disponibilizados para replicação mediante solicitação razoável dirigida ao autor correspondente. O estudo foi aprovado pelo Comitê de Ética em Pesquisa da Universidade Federal de Minas Gerais (UFMG) sob número CAAE 37228120.9.0000.5149 e pelos conselhos de saúde locais. O estudo PROVAR+ é um programa de rastreamento cardiovascular estabelecido em 2014, como uma colaboração internacional entre a UFMG, a Rede de Telessaúde de Minas Gerais^8^ e o Children's National Health System, Washington, DC, Estados Unidos. O presente subestudo foi realizado entre fevereiro de 2022 e maio de 2023 em Divinópolis, localizada na região central de Minas Gerais, no Sudeste do Brasil. A cidade possui 231.091 habitantes e Índice de Desenvolvimento Humano médio de 0,764 ( Figura Suplementar 1 ).

O estudo PROVAR+ utiliza não especialistas para aquisição de imagens por meio de transferência de tarefas ( *task-shifting* ), em dispositivos portáteis (VScan® Extend, GE Healthcare, Milwaukee, Wisconsin, Estados Unidos) para detecção ecocardiográfica de DC na AP e interpretação remota por especialistas no Brasil e Estados Unidos segundo critérios da Sociedade Americana de Ecocardiografia (ASE).^10^ Os centros de AP que participaram do estudo foram incluídos de acordo com as prioridades das autoridades de saúde, com base em baixos índices socioeconômicos (considerando o Índice de Desenvolvimento Humano) e acesso limitado à atenção cardiovascular secundária e terciária, com longas filas para ecocardiografia padrão eletiva.

### Implementação e treinamento

Durante a fase de implementação, 6 profissionais de saúde (3 enfermeiros, 3 técnicos de enfermagem) em 16 centros de AP passaram por um processo educacional misto sobre ecocardiografia, composto por 9 módulos on-line padronizados (disponíveis em: http://www.wiredhealthresources.net/EchoProject/index.html ) e foram treinados em protocolos ecocardiográficos simplificados no Hospital Universitário da UFMG, utilizando máquinas portáteis, realizando em média 32 horas de treinamento prático. Localmente, um currículo educacional para pacientes/comunidades sobre DC foi implementado pela equipe do estudo, agentes comunitários de saúde e estudantes de medicina, durante visitas regulares e em atividades de grupo programadas (conhecidas como Grupos Operacionais), usando *flipcharts* educacionais impressos projetados para o estudo, como uma colaboração com a Reach Foundation, Cidade do Cabo, África do Sul ( Figura Suplementar 2 ).

### Critérios de inclusão

Todos os pacientes adultos (≥ 18 anos) de ambos os sexos que se apresentaram nos centros de AP participantes para consultas agendadas ou não agendadas, que foram submetidos ao tele-ECG com base nas indicações clínicas da equipe responsável, eram potencialmente elegíveis e foram inscritos prospectivamente com base nos resultados do ECG, após assinatura do termo de consentimento livre e esclarecido.

Os ECGs foram captados por equipamentos comerciais vinculados a software proprietário específico, que possibilita a obtenção do sinal de ECG e dos dados clínicos, e transmitidos via internet para um servidor central do Centro de Telessaúde da UFMG. O profissional de saúde solicitante coletou histórico inicial, informações demográficas e dados clínicos. Os ECGs foram analisados centralmente por uma equipe de cardiologistas experientes, utilizando software específico semiautomatizado com ferramentas de medição e ampliação, com inspeção visual e subsequente classificação pelo código de Minnesota. O código de Minnesota é o sistema de classificação de ECG mais utilizado no mundo. Foi desenvolvido na década de 1950 por Dr. Henry Blackburn e utiliza um conjunto definido de regras de medição para atribuir códigos numéricos específicos de acordo com a gravidade dos achados.^11 , 12^ Na presença de uma discrepância entre os relatórios automatizados e a interpretação do cardiologista, os exames foram auditados para classificação final.

**Figura 1:**
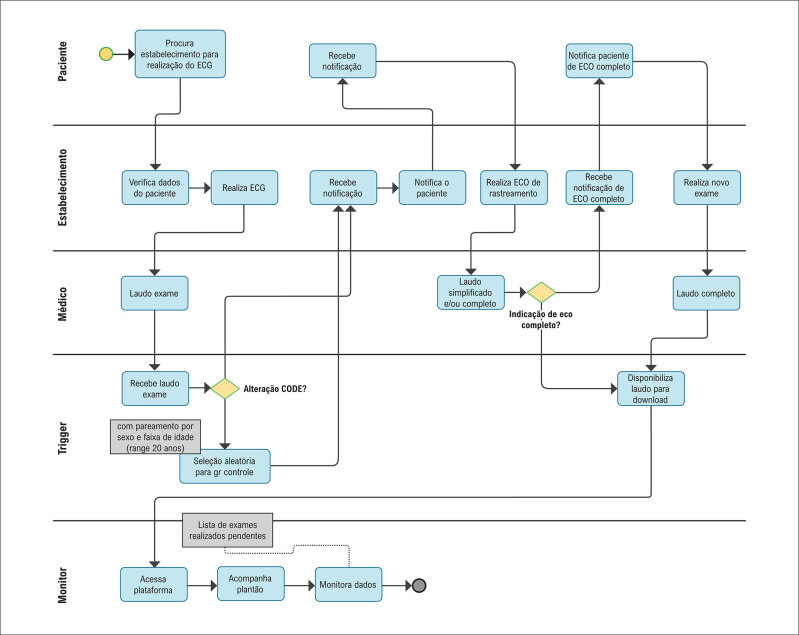
Sistema de gatilho desenvolvido para o estudo, baseado no sistema proprietário de tele-eletrocardiografia (SigTel, Universidade Federal de Minas Gerais, Belo Horizonte, Brasil), sinalizando exames com anormalidades maiores pelo código de Minnesota, acionando o ecocardiograma de rastreamento com interpretação remota por cardiologistas credenciados.

Por meio de um sistema proprietário de gatilhos desenvolvido para o projeto ( Figura 1 ), todos os pacientes com grandes anormalidades no ECG pelo código de Minnesota, após o diagnóstico final do ECG ( Tabela Suplementar 1 ), e uma amostra aleatória 1:5 de indivíduos sem anormalidades foram sinalizados na interface de usuário da equipe para inclusão no estudo. Os pacientes consentidos foram então agendados para uma consulta para aplicação de um questionário clínico, seguida de ecocardiograma de rastreamento realizado por não médicos (GE VScan Extend^®^), que foi remotamente interpretada exclusivamente por cardiologistas credenciados.

### Procedimentos de ecocardiograma de rastreamento

**Figura 2:**
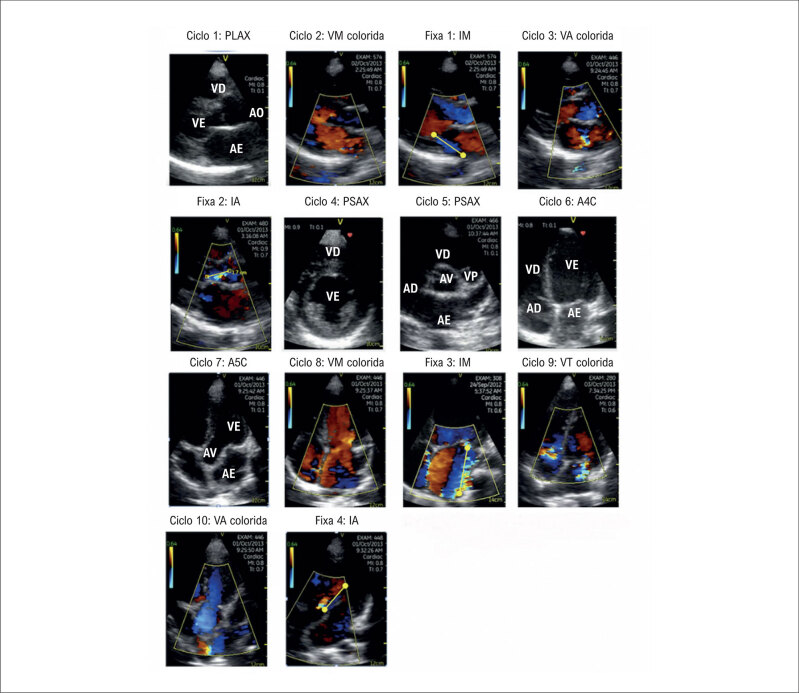
Protocolo de rastreamento simplificado para dispositivos portáteis de avaliação de doença cardíaca em adultos, composto por 14 imagens (7 visualizações): 10 ciclos e 4 fixas. A4C: apical de quatro câmaras; A5C: apical de cinco câmaras; AD: átrio direito; AE: átrio esquerdo; AO: aorta; IA: insuficiência aórtica; IM: insuficiência mitral; PLAX: eixo paraesternal longo; PSAX: eixo paraesternal curto; VA: valva aórtica; VD: ventrículo direito; VE: ventrículo esquerdo; VM: valva mitral; VP: valva pulmonar; VT: valva tricúspide.

Foi aplicado um protocolo de 7 visualizações de ponto de cuidado ( *point-of-care* ) para rastreamento, com foco na avaliação valvar e na morfologia e função ventricular esquerda/direita, além do derrame pericárdico ( Figura 2 ), utilizando dispositivos portáteis (GE VScan Extend^®^). Foram aplicados os critérios diagnósticos da ASE,^13^ com exceção das medidas do Doppler espectral, ausentes nesses aparelhos. A DC significativa foi definida como doença valvar moderada a grave (regurgitação ou estenose), disfunção/hipertrofia ventricular, DC congênita, derrame pericárdico ou qualquer anormalidade da motilidade^10^ ( Tabela Suplementar 1 ). Foi recomendada a repetição do rastreamento para aqueles com qualidade de imagem subótima. Para interpretação, imagens DICOM foram carregadas em um ambiente de computação em nuvem proprietária (SigTel®, UFMG, Belo Horizonte, Minas Gerais, Brasil) para relatórios online utilizando software de imagem disponível comercialmente,^14^ no Brasil e nos Estados Unidos (CAS, MCN, CS). Todos os relatórios foram disponibilizados online para os centros de AP, informando que não se tratava de um diagnóstico final.

### Análise estatística

Os dados foram inseridos na nuvem proprietária SigTel® e exportados para o banco de dados RedCap®.^15^ A análise estatística foi realizada utilizando o software SPSS® versão 23.0 para Mac OSX (SPSS Inc., Chicago, Illinois, Estados Unidos). Por ser um estudo exploratório, não foi realizado nenhum cálculo amostral pré-especificado e foram incluídos todos os indivíduos elegíveis submetidos ao rastreamento por tele-ECG em 17 meses. Foi usado o teste de Shapiro-Wilk para avaliar a distribuição das variáveis contínuas. As variáveis categóricas, expressas em números e porcentagens, foram comparadas entre os grupos (com e sem DC grave no ecocardiograma de rastreamento), utilizando o teste exato de Fisher, enquanto os dados contínuos, expressos em mediana e Q1/Q3 (25%/75%), foram comparados usando o teste U de Mann-Whitney, conforme apropriado.

Foi utilizada a regressão logística multivariada para analisar a associação entre a presença de anormalidades maiores no ECG, de acordo com o código de Minnesota, e a presença de DC grave no ecocardiograma de rastreamento. As variáveis significativas (p < 0,10) nas análises univariadas foram incluídas nos modelos multivariados. Foram ajustados 4 modelos de regressão da seguinte forma: 1) modelo não ajustado; 2) modelo 1 ajustado por idade e sexo; 3) modelo 2 mais fatores de risco (hipertensão e diabetes); 4) modelo 3 mais história de doença cardiovascular (Chagas, cardiopatia reumática, cardiopatia isquêmica, AVC e insuficiência cardíaca). Todas as variáveis clínicas consideradas foram coletadas durante a entrevista clínica. Para todas as análises, foi considerado estatisticamente significativo um nível de significância bicaudal de 0,05.

## Resultados

Após o período inicial de treinamento, a qualidade dos exames adquiridos foi classificada como satisfatória para interpretação em > 90% dos 50 casos iniciais durante o período de avaliação de qualidade, e então o recrutamento dos pacientes foi iniciado.

No total, 1.411 pacientes foram submetidos ao ecocardiograma de rastreamento em 17 meses. De acordo com o código de Minnesota, 1.149 (81%) apresentaram anormalidades maiores no ECG e 19% foram incluídos no grupo controle. A idade mediana foi de 67 anos (intervalo interquartil de 60 a 74); 51,4% eram do sexo masculino; 76,3% tinham hipertensão, 37,7% diabetes e 22,3% cardiopatia isquêmica. Embora as taxas de insuficiência cardíaca preexistente, doença de Chagas e cardiopatia reumática tenham sido globalmente baixas, as variáveis clínicas e demográficas retratam um perfil de alto risco da amostra. No geral, os pacientes com DC grave na ecocardiografia eram mais velhos e apresentavam maior prevalência de hipertensão e diabetes, bem como taxas mais altas de insuficiência cardíaca e cardiopatia isquêmica previamente conhecidas ( Tabela 1 ).

**Tabela 1:** Características da amostra incluída e comparação de variáveis demográficas e clínicas entre pacientes com e sem doença cardíaca grave detectada por ecocardiograma de rastreamento

Característica		N	Geral^1^	Anormalidade maior no eco (n=475)	Normal (n=936)	Valor p^2^
Sexo		1.411				0,092
	Feminino		686 (48,6%)	216 (45,5%)	470 (50,2%)	
	Masculino		725 (51,4%)	259 (54,5%)	466 (49,8%)	
Idade (anos)		1.411	67,0 (59,5; 74,0)	73,0 (64,0; 80,0)	64,0 (58,0; 71,0)	**<0,001**
ECG		1.411				<0,001
	Anormalidade maior		1.149 (81,4%)	424 (89,3%)	725 (77,5%)	
	Normal		262 (18,6%)	51 (10,7%)	211 (22,5%)	
Eco de acompanhamento imediatamente recomendado		1.411	368 (26,1%)	334 (70,3%)	34 (3,6%)	**<0,001**
Hipertensão		1.411	1.076 (76,3%)	397 (83,6%)	679 (72,5%)	**<0,001**
Diabetes		1.411	532 (37,7%)	197 (41,5%)	335 (35,8%)	**0,037**
Doenças de Chagas		1.411	13 (0,9%)	6 (1,3%)	7 (0,7%)	0,381
Insuficiência cardíaca		1.411	38 (2,7%)	27 (5,7%)	11 (1,2%)	**<0,001**
IAM/doença arterial coronariana		1.411	314 (22,3%)	142 (29,9%)	172 (18,4%)	**<0,001**
Cardiopatia reumática		1.411	4 (0,3%)	1 (0,2%)	3 (0,3%)	>0,999
AVC		1.411	78 (5,5%)	32 (6,7%)	46 (4,9%)	0,157

1 n (%); mediana (intervalo interquartil).

2 Teste qui-quadrado de Pearson; teste de soma de postos de Wilcoxon; teste exato de Fisher. AVC: acidente vascular cerebral; ECG: eletrocardiograma; eco: ecocardiograma; IAM: infarto agudo do miocárdio.

Um total de 475 (33,7%) pacientes apresentaram DC grave no ecocardiograma de rastreamento. Entre aqueles com anormalidades maiores no ECG, 37% (N = 424) apresentaram ecocardiograma anormal, em comparação com 19% (N = 51) daqueles sem achados maiores no ECG (p < 0,001). Após avaliação por consenso das imagens de rastreamento, foi recomendado ecocardiograma de acompanhamento prioritário para 334 pacientes (70,3%) com DC grave no rastreamento e para 34 (3,6%) daqueles sem anormalidades, para esclarecimento diagnóstico ou devido a imagens subótimas.

Nos modelos de regressão, as anormalidades maiores do ECG pelo código de Minnesota se associaram a uma chance 2,4 vezes maior de se ter DC grave no ecocardiograma de rastreamento na análise bivariada e permaneceram significativas (p < 0,001) após ajustes no modelo 2 (OR = 2,57), modelo 3 (OR = 2,52) e modelo 4 (OR = 2,23) ( Tabela 2 ). Apesar dos múltiplos ajustes, a associação entre anormalidades no ECG e no ecocardiograma permaneceu forte no modelo 4, com OR > 2,0 e valor de p < 0,001 ( Tabela 2 ).

**Tabela 2:** Modelos (4) de regressão ajustados para avaliar a associação entre as anormalidades maiores no tele-ECG pelo código de Minnesota e a presença de doença cardíaca grave detectada por ecocardiograma de rastreamento

Variáveis/modelo		OR (IC 95%)	Valor p
**Modelo 1**			
	(Intercepto)	0,24 (0,18-0,33)	**< 0,001**
	Anormalidade maior no ECG	2,42 (1,76-3,39)	**< 0,001**
**Modelo 2**			
	(Intercepto)	0 (0-0)	**< 0,001**
	Anormalidade maior no ECG	2,57 (1,84-3,65)	**< 0,001**
	Sexo	1,37 (1,08-1,74)	**0,009**
	Idade	1,07 (1,06-1,08)	**< 0,001**
**Modelo 3**			
	(Intercepto)	0 (0-0)	**< 0,001**
	Anormalidade maior no ECG	2,52 (1,80-3,58)	**< 0,001**
	Sexo	1,41 (1,11-1,79)	**0,005**
	Idade	1,07 (1,06-1,08)	**< 0,001**
	Hipertensão	1,46 (1,07-2,00)	**0,017**
	Diabetes	1,05 (0,82-1,35)	0,688
**Modelo 4**			
	(Intercepto)	0 (0-0)	**< 0,001**
	Anormalidade maior no ECG	2,23 (1,59-3,19)	**< 0,001**
	Sexo	1,35 (1,06-1,73)	**0,015**
	Idade	1,07 (1,06-1,09)	**< 0,001**
	Hipertensão	1,32 (0,97-1,81)	0,083
	Diabetes	1,03 (0,80-1,33)	0,825
	Doença de Chagas	1,09 (0,29-3,82)	0,897
	Insuficiência cardíaca	5,18 (2,43-11,87)	**< 0,001**
	IAM/doença arterial coronariana	1,56 (1,18-2,07)	**0,002**
	Cardiopatia reumática (conhecida)	0,37 (0,01-6,16)	0,491
	AVC	1,18 (0,72-1,93)	0,504

AVC: acidente vascular cerebral; ECG: eletrocardiografia; IAM: infarto agudo do miocárdio; IC: intervalo de confiança; OR: odds ratio.

Dentre as variáveis demográficas e clínicas consideradas para os modelos, idade (a cada 1 ano), sexo masculino e história prévia de insuficiência cardíaca e de infarto agudo do miocárdio/doença arterial coronariana foram preditores independentes de DC grave no ecocardiograma de rastreamento, assim como as anormalidades maiores no ECG ( Tabela 2 ).

## Discussão

Nosso estudo de prova de conceito mostrou que uma combinação de ferramentas de telecardiologia pode ser uma estratégia promissora para rastreamento de DC e priorização de referenciamento para exames no ambiente da AP. Anormalidades maiores no tele-ECG (um recurso de telessaúde difundido no Brasil) adequadamente prediz a presença de DC grave no ecocardiograma de rastreamento com interpretação remota, independentemente das variáveis clínicas. Esses achados apontam para a utilidade de tais ferramentas para melhorar os cuidados cardiovasculares em regiões com poucos recursos, através da potencial incorporação de modalidades disponíveis e de baixo custo nos escores de risco clínico. No Brasil, o acesso aos cuidados de saúde é desigual,^16^ e as taxas de mortalidade associadas às doenças cardiovasculares também variam dependendo das características regionais e das condições socioeconômicas.^1^ Portanto, a descentralização dos serviços de saúde é um desafio e uma necessidade crescente no país, com a finalidade de fornecer melhor acesso aos cuidados de saúde para a população necessitada.

Nesse sentido, além de melhorar a eficiência e reduzir os custos, a telemedicina pode ampliar as fronteiras da AP, proporcionando acesso à saúde para populações remotas e potencialmente ampliando a atuação dos profissionais de saúde, integrando-os aos serviços de saúde especializados localizados em hospitais e centros de referência e, em última instância, democratizando o acesso à prevenção, diagnóstico e tratamento.^8 , 17^ No entanto, sabe-se que o Brasil apresenta inúmeros desafios estruturais para alcançar o acesso universal a dispositivos de telessaúde, especialmente relacionados ao acesso adequado à conexão à internet. Essa situação tende a ser ainda pior em regiões remotas e periféricas, representando uma barreira significativa para a disseminação e consolidação da telemedicina no país.^1 , 2^ Ainda que a infraestrutura técnica para a telemedicina seja resolvida no Brasil, ainda haverá risco de escassez de capacidade local e de pessoal para promover a expansão do acesso aos cuidados de saúde, dado que os serviços remotos são essencialmente inter e multidisciplinares.^2^ Assim, a consolidação de parcerias entre os setores público e privado é um passo essencial para tornar os recursos de telessaúde disponíveis e efetivamente funcionais em múltiplas localidades, dadas as dimensões continentais do país.

Visando abordar essas questões, programas governamentais implementaram o tele-ECG como uma das ferramentas prioritárias de telessaúde em diversas regiões do país nas últimas décadas,^8 , 18^ aproveitando parcerias entre a AP de vários municípios brasileiros e hospitais universitários, onde o traçado do ECG é interpretado em tempo real e os laudos são imediatamente enviados de volta às unidades básicas.^8^ O tele-ECG tem se mostrado uma ferramenta robusta de telessaúde para localidades distantes dos grandes centros urbanos e com acesso limitado a serviços de saúde especializados.^8 , 9^ Os dados disponíveis consolidaram-no como uma ferramenta válida e custo-efetiva para melhorar a acurácia diagnóstica e terapêutica em locais onde faltam especialistas, além de promover cuidados de saúde para populações com acesso subótimo.^9 , 19^ Nossos dados reforçam a utilidade diagnóstica do tele-ECG de uma perspectiva mais ampla, com previsão ideal de DC grave detectada pelo ecocardiograma de rastreamento, mesmo após ajuste para variáveis clínicas relevantes.

Nesse cenário, a combinação de ferramentas de telecardiologia para estratificação de risco cardiovascular na AP surge como uma estratégia promissora, considerando a possibilidade de integração do ecocardiograma de rastreamento realizada por não médicos com interpretação remota por cardiologistas credenciados nos cuidados de rotina.^6 , 20^ Vale a pena destacar que o ecocardiograma de rastreamento com dispositivos portáteis, conforme testado em nosso protocolo, não é proposto como uma ferramenta de diagnóstico final, com avaliação detalhada de variáveis morfofuncionais; em vez disso, destina-se à aquisição rápida de protocolos ultrassonográficos simplificados por uma equipe não médica com rápido treinamento técnico, visando sinalizar anormalidades importantes para fins de triagem.^21 – 23^ Indivíduos com achados anormais devem idealmente ser submetidos a um ecocardiograma padrão confirmatório com um protocolo abrangente, focado na avaliação mais profunda das alterações morfofuncionais.^21^ Essencialmente, protocolos de rastreamento mais simples e rápidos são mais fáceis de serem replicados por pessoal não médico brevemente treinado para o posicionamento da sonda, potencialmente facilitando a implementação da estratégia para mais regiões, integrada com a AP.^6 , 24^ No Brasil, no entanto, essa abordagem de transferência de tarefas só é permitida em protocolos de pesquisa, pois imagens de ultrassom só podem ser adquiridas por médicos credenciados. As discussões sobre tais regulamentações com os decisores políticos e conselhos médicos são necessárias para permitir a expansão da estratégia, se for comprovadamente viável e custo-efetiva, para outros fins que não a pesquisa.

Estudos focados na cardiopatia reumática demonstraram que a estratégia de rastreamento é eficaz para o diagnóstico precoce^22 , 23^ e reconhecidamente melhora o prognóstico.^25^ O ecocardiograma de rastreamento é geralmente mais sensível do que específico para detecção de cardiopatia reumática latente.^21 , 24^ Para outras patologias cardíacas, os achados do rastreamento têm uma correlação significativa com o ecocardiograma padrão realizado por especialistas.^21^ Embora existam razões reconhecidas para as discrepâncias entre os achados dos ecocardiogramas de rastreamento e padrão,^10 , 21^ a experiência e o histórico de treinamento dos operadores, além dos recursos limitados do dispositivos de rastreamento ultraportáteis, em comparação com máquinas padrão totalmente funcionais, são fatores adicionais que explicam as discordâncias. Assim, os investimentos na educação, treinamento e garantia de qualidade do pessoal e no desenvolvimento tecnológico colaborativo são abordagens fundamentais para a obtenção de melhores resultados.^21^

Considerando isso, a associação entre os achados do tele-ECG e DC grave detectada pelo ecocardiograma de rastreamento com leitura remota levanta a possibilidade de combinar tais ferramentas no futuro, otimizando a utilização dos recursos de saúde, evitando custos de transporte e racionalizando encaminhamentos dos níveis de AP para secundária. Nossos resultados sugerem que a associação entre anormalidades maiores no tele-ECG e no tele-eco é independente dos perfis demográfico e clínico dos pacientes, conforme indicado pelos múltiplos modelos ajustados, reforçando a hipótese de que uma possível combinação desses testes permitiria uma estratificação de risco mais precisa na AP.^5 , 7^ Além disso, a possibilidade de pré-rastrear os pacientes no centro de saúde primário sempre que um ecocardiograma for solicitado pelo médico assistente, por meio de um questionário clínico, tele-ECG de triagem e, quando indicado, ecocardiograma de rastreamento, seria uma maneira viável de prever com acurácia a presença de DC significativa, racionalizar as indicações de ecocardiograma padrão e evitar atrasos longos e às vezes desnecessários no encaminhamento.^26^ A viabilidade de integrar o ecocardiograma de rastreamento na AP já foi demonstrado anteriormente por nosso grupo, bem como o potencial para fornecer diagnóstico precoce de DC para adultos e idosos.^5 , 6^

O cenário da atenção secundária pública brasileira é desafiador na maioria dos estados, com longas demoras para exames e consultas cardiovasculares complementares, principalmente em áreas de difícil acesso, municípios pequenos e regiões distantes dos grandes centros urbanos.^1 , 2^ Longos tempos de espera frequentemente representam uma demora para início e otimização do tratamento. Neste contexto, uma estratégia de estratificação de risco mais precisa pode ser útil tanto para adaptar o tratamento do paciente quanto para reduzir a demanda para encaminhamentos, em última instância resultando em listas de espera mais curtas. Com base nos nossos resultados, a possibilidade de pré-triagem com tele-ECG já é uma realidade, com resultados iniciais convincentes. Além disso, a possibilidade de diagnóstico semiautomático de ECG, já disponível na Rede de Telessaúde de Minas Gerais,^18^ e algoritmos de aprendizado de máquina para ECG^27^ e ecocardiografia^28 , 29^ podem aumentar ainda mais o poder preditivo dos métodos e reduzir os custos operacionais.

Uma questão que permanece inexplorada é a possibilidade de desenvolver um escore de predição combinando variáveis clínicas simples, tele-ECG e ecocardiograma de rastreamento com leitura remota. Embora a adição de ecocardiograma de rastreamento às variáveis clínicas melhore reconhecidamente o poder preditivo de um escore de risco existente,^5^ o efeito da inclusão do tele-ECG no modelo e o impacto prognóstico da estratégia ainda são desconhecidos. Mais estudos são necessários para definir protocolos otimizados de ecocardiografia de rastreamento (por exemplo, protocolo simplificado versus projeção única), bem como para estabelecer indicações e cenários de rastreamento ideais para otimização da busca ativa de casos. Por fim, são necessárias discussões com conselhos médicos e autoridades de saúde à luz dos dados emergentes sobre esse tema, considerando que a aquisição de imagens por não médicos não é permitida fora de projetos de pesquisa no Brasil e em outros países em desenvolvimento, mesmo com interpretação exclusiva por cardiologistas. As restrições corporativistas podem ser uma barreira para a implementação de estratégias de redução de custos na área cardiovascular.

### Limitações

Nosso estudo apresenta algumas limitações. Em primeiro lugar, foi um estudo de implementação de prova de conceito, no qual foi avaliada apenas a acurácia do tele-ECG, em oposição à sua incorporação em um escore de risco abrangente. No entanto, uma avaliação gradual da acurácia dos testes individuais é um passo crucial para o desenvolvimento de escores preditivos, conforme planejado para as fases posteriores do estudo PROVAR+. Em segundo lugar, o padrão-ouro para definição de DC grave foi o ecocardiograma de rastreamento com dispositivos portáteis, o que pode ter levado a limitações diagnósticas. Além disso, essa geração do GE-VSCAN Extend não possui recursos de Doppler espectral. Toda a interpretação, entretanto, foi feita consensualmente por pelo menos 2 cardiologistas experientes, com expertise em projetos de pesquisa relacionados ao rastreamento. Em terceiro lugar, não foram realizados procedimentos de amostragem estratificada e o recrutamento foi feito consecutivamente por conveniência com base no sistema de tele-ECG, limitando a extrapolação dos achados para a população brasileira. Além disso, os dados do ecocardiograma de rastreamento de pacientes não elegíveis para rastreamento, com base nos resultados do ECG, não estão disponíveis para comparação. Finalmente, o tamanho da amostra limita uma visão mais profunda sobre associações específicas entre anormalidades individuais no ECG e achados funcionais/morfológicos do ecocardiograma. Apesar das limitações supracitadas, até onde sabemos, este é o maior estudo realizado na América Latina com o objetivo de avaliar uma abordagem combinada de ferramentas de telecardiologia na AP, e nossos dados apontam para a necessidade de avaliações adicionais, conforme planejado para um futuro próximo.

## Conclusões

As anormalidades do tele-ECG aumentaram a probabilidade de doença cardíaca grave no ecocardiograma de rastreamento, mesmo após ajustes para variáveis demográficas e clínicas. A combinação de ferramentas de telecardiologia e dados clínicos pode melhorar a estratificação de risco na AP, e os resultados apontam para o desenvolvimento de escores de risco abrangendo diversas modalidades de priorização de cuidados.

## *Material suplementar

Para tabelas adicionais, por favor, clique aqui .

Para figuras adicionais, por favor, clique aqui .
