# Supplementary material for: Combinação de Ferramentas de Telecardiologia para Estratificação de Risco Cardiovascular na Atenção Primária: Dados do Estudo PROVAR+
Source: Arq Bras Cardiol. 2024 Mar 21;121(2):e20230653. [Article in Portuguese] doi: 10.36660/abc.20230653 (PMC12092040; doi:10.36660/abc.20230653)
Supplement: Translation

# Translation: Combination of Tele-Cardiology Tools for Cardiovascular Risk Stratification in Primary Care: Data from the PROVAR+ Study

**Authors:** Lucas Leal Fraga, Bruno Ramos Nascimento, Beatriz Costa Haiashi, Alexandre Melo Ferreira, Mauro Henrique Agapito Silva, Isabely Karoline da Silva Ribeiro, Gabriela Aparecida Silva, Wanessa Campos Vinhal, Mariela Mata Coimbra, Cássia Aparecida Silva, Cristiana Rosa Lima Machado, Magda C. Pires, Marina Gomes Diniz, Luiza Pereira Afonso Santos, Arthur Maia Amaral, Lucas Chaves Diamante, Henrique Leão Fava, Craig Sable, Maria Carmo Pereira Nunes, Antonio Luiz P. Ribeiro, Clareci Silva Cardoso

**Affiliations:** 1 Belo Horizonte MG Brazil Hospital das Clínicas da Universidade Federal de Minas Gerais – Serviço de Cardiologia e Cirurgia Carvdiovascular, Belo Horizonte, MG – Brazil; 2 Belo Horizonte MG Brazil Hospital Madre Teresa – Serviço de Hemodinâmica, Belo Horizonte, MG – Brazil; 3 Belo Horizonte MG Brazil Universidade Federal de Minas Gerais – Departamento de Clínica Médica – Faculdade de Medicina, Belo Horizonte, MG – Brazil; 4 Belo Horizonte MG Brazil Hospital das Clínicas da Universidade Federal de Minas Gerais – Centro de Telessaúde, Belo Horizonte, MG – Brazil; 5 Divinópolis MG Brazil Universidade Federal de São João del Rei – Campus Centro-Oeste Dona Lindu – Campus Divinópolis, Divinópolis, MG – Brazil; 6 Belo Horizonte MG Brazil Universidade Federal de Minas Gerais - Instituto de Ciências Exatas – Departamento de Estatística, Belo Horizonte, MG – Brazil; 7 Belo Horizonte MG Brazil Faculdade de Ciências Médicas de Minas Gerais – Faculdade de Medicina, Belo Horizonte, MG – Brazil; 8 Ouro Preto MG Brazil Universidade Federal de Ouro Preto – Departamento de Medicina, Ouro Preto, MG – Brazil; 9 Washington Columbia USA Children's National Health System – Cardiology, Washington, District of Columbia – USA

**Keywords:** Cardiovascular Diseases, Mass Screening, Telemedicine, Electrocardiography, Risk Grade

## Abstract

**Background:**

Tele-cardiology tools are valuable strategies to improve risk stratification.

**Objective:**

We aimed to evaluate the accuracy of tele-electrocardiography (ECG) to predict abnormalities in screening echocardiography (echo) in primary care (PC).

**Methods:**

In 17 months, 6 health providers at 16 PC units were trained on simplified handheld echo protocols. Tele-ECGs were recorded for final diagnosis by a cardiologist. Consented patients with major ECG abnormalities by the Minnesota code, and a 1:5 sample of normal individuals underwent clinical questionnaire and screening echo interpreted remotely. Major heart disease was defined as moderate/severe valve disease, ventricular dysfunction/hypertrophy, pericardial effusion, or wall-motion abnormalities. Association between major ECG and echo abnormalities was assessed by logistic regression as follows: 1) unadjusted model; 2) model 1 adjusted for age/sex; 3) model 2 plus risk factors (hypertension/diabetes); 4) model 3 plus history of cardiovascular disease (Chagas/rheumatic heart disease/ischemic heart disease/stroke/heart failure). P-values < 0.05 were considered significant.

**Results:**

A total 1,411 patients underwent echo; 1,149 (81%) had major ECG abnormalities. Median age was 67 (IQR 60 to 74) years, and 51.4% were male. Major ECG abnormalities were associated with a 2.4-fold chance of major heart disease on echo in bivariate analysis (OR = 2.42 [95% CI 1.76 to 3.39]), and remained significant after adjustments in models (p < 0.001) 2 (OR = 2.57 [95% CI 1.84 to 3.65]), model 3 (OR = 2.52 [95% CI 1.80 to3.58]), and model 4 (OR = 2.23 [95%CI 1.59 to 3.19]). Age, male sex, heart failure, and ischemic heart disease were also independent predictors of major heart disease on echo.

**Conclusions:**

Tele-ECG abnormalities increased the likelihood of major heart disease on screening echo, even after adjustments for demographic and clinical variables.

**Figure:** ECG: electrocardiography; ECHO: echocardiogram.

## Introduction

Brazil faces a high incidence of cardiovascular disease as the main cause of mortality, a pattern similar to that observed in developed countries.^1^However, as it is a developing nation with a single, universal healthcare system (Unified Health System, abbreviated SUS in Portuguese), significant challenges arise in the search for a balance between prevention and treatment, in an epidemiological scenario that merges characteristics of developed and developing countries.^2^Hence, it is essential to adopt effective strategies to improve the allocation of health resources, with a focus on prevention. Reallocation of resources to priority areas requires a strategic approach that requires both technology and medical knowledge.

A crucial tool for cardiovascular screening is the echocardiogram (echo), which makes it possible to detect, classify, and stratify the risk of structural heart disease (HD). Thanks to developing technology, echo has progressively become portable and accessible, moving from imaging centers to the point-of-care, with a potential to reach unserved settings.^3 , 4^Therefore, a two-step approach, which includes the performance of echo by non-experts, followed by remote interpretation by certified cardiologists, is a promising screening strategy.^5 , 6^This may enable more effective resource allocation and implementation of broader screening programs, allowing for earlier diagnosis and prompt treatment, especially where resources are scarce.

Currently, the Brazilian SUS allows primary care (PC) physicians to request echo for any diagnostic purposes, and patients are prioritized based only on the date of request. Long queues and the lack of a prioritization system for more urgent cases frequently lead to delayed diagnosis, contributing to unfavorable outcomes.^5 , 6^In this setting, given that local health systems have an extremely limited availability of specialized tests and referrals for secondary care, long waiting times are commonly observed. Furthermore, referrals of patients without significant clinical abnormalities for specialized tests are frequent, contributing to long delays.^7^In an attempt to solve this problem, it has been demonstrated that the addition of screening echo to a clinical risk stratification score may be a promising tool for prioritizing referrals for conventional echo and cardiology appointments, which can potentially result in a reduction of waiting lists in underserved areas and better allocation of health resources.^5 , 7^

An important tool for expanding portable echo screening is telemedicine, as it allows for prompt remote interpretation by specialists, markedly in regions with greater social vulnerability.^5 – 7^The Telehealth Network of Minas Gerais was implemented in 2005 by the Government of the State of Minas Gerais, Brazil, aimed at connecting university hospitals to local health services, offering support to health professionals through tele-electrocardiography (ECG), tele-assistance, and, more recently, tele-echo, in addition to providing support through teleconsultations.^8^The tele-ECG system is now available throughout the country, with a recent expansion to other South American nations.^9^In this study, we aimed to evaluate the accuracy of tele-ECG in remote sites to predict abnormalities in screening echo in Brazilian PC, as a combined strategy for risk stratification.

## Methods

The procedures and methods of this study will be made available for replication upon reasonable request directed to the corresponding author. The Institutional Review Board of Universidade Federal de Minas Gerais (UFMG) approved the study under CAAE number 37228120.9.0000.5149 and by the local boards of health. The PROVAR+ study is a cardiovascular screening program established in 2014, as an international collaboration between UFMG, the Telehealth Network of Minas Gerais^8^and the Children's National Health System, Washington, DC, United States. This sub study took place between February 2022 and May, 2023 in the city of Divinópolis, located in the central region of the state of Minas Gerais, in the Southeast Region of Brazil. The city has 231,091 inhabitants and mean Human Development Index of 0.764 ( Supplement Figure 1 ).

The PROVAR+ study utilizes non-experts for image acquisition through task-shifting, on handheld devices (VScan® Extend, GE Healthcare, Milwaukee, Wisconsin, United States) for echocardiographic detection of HD in the PC setting and remote interpretation by experts in Brazil and the United States, according to the American Society of Echocardiography (ASE)^10^criteria. The PC centers participating in the study were included according to the priorities of health authorities, based on low socioeconomic indexes (considering the Human Development Index) and limited access to secondary and tertiary cardiovascular care, with long queues for elective standard echo.

### Implementation and training

During the implementation phase, 6 healthcare workers (3 nurses, 3 nursing technicians) at 16 PC centers underwent a mixed educational process on echo, consisting of 9 standardized online modules (available at: http://www.wiredhealthresources.net/EchoProject/index.html ) and were trained on simplified echo protocols at the UFMG University Hospital, utilizing handheld machines, performing an average of 32 hours of hands-on training. Locally, a patient/community educational curriculum on HD was deployed by the study staff, community health agents, and medical students, during regular visits and in scheduled group activities (known as Operational Groups), using educational printed flipcharts designed for the study, as a collaboration with the Reach Foundation, Cape Town, South Africa ( Supplement Figure 2 ).

### Inclusion criteria

All adult patients (≥ 18 years) of both sexes presenting to the participant PC facilities for scheduled or non-scheduled appointments, who were submitted to the tele-ECG based on clinical indications by the attending team, were potentially eligible and prospectively enrolled based on the ECG results, after signing the informed consent form.

The ECGs were captured by commercial equipment linked to specific proprietary software, which makes it possible to obtain ECG signal and clinical data, and transmitted by internet to a central server at the UFMG Telehealth Center. The requesting healthcare provider collected baseline history, demographic information, and clinical data. ECGs were centrally analyzed by a team of experienced cardiologists, utilizing specific semi-automated software with measurement and magnification tools, with visual inspection and subsequent classification by the Minnesota code, which is the most widely used ECG classification system worldwide. It was developed in the 1950s by Dr. Henry Blackburn, and it utilizes a defined set of measurement rules to assign specific numerical codes according to the severity of findings.^11 , 12^In the presence of a discrepancy between automated reports and the cardiologist's interpretation, exams were audited for a final classification.

Through a proprietary trigger system developed for the project ( Figure 1 ), all patients with major ECG abnormalities by the Minnesota code, after the final ECG diagnosis ( Supplement Table 1 ), and a random 1:5 sample of individuals without abnormalities were flagged in the team's user interface for inclusion in the study. Consented patients were then scheduled for an appointment for application of a clinical questionnaire, followed by echo screening performed by non-physicians (GE VScan Extend®), which was remotely interpreted exclusively by certified cardiologists.

**Figure 1:** Trigger system developed for the study, based on the proprietary tele-electrocardiography system (SigTel, Universidade Federal de Minas Gerais, Belo Horizonte, Brazil), flagging exams with major abnormalities by the Minnesota code, triggering the screening echocardiogram with remote interpretation by certified cardiologists. ECG: electrocardiogram; echo: echocardiogram.

### Screening echocardiography procedures

A point-of-care 7-view protocol was applied for screening, focusing on valvular assessment and left/right ventricular morphology and function, in addition to pericardial effusion ( Figure 2 ), utilizing handheld devices (GE VScan Extend®). The ASE diagnostic criteria^13^were applied, with the exception of spectral Doppler measurements, which were absent in these devices. Significant HD was defined as moderate to severe valve disease (regurgitation or stenosis), ventricular dysfunction/hypertrophy, congenital heart disease, pericardial effusion, or any wall-motion abnormalities^10^( Supplement Table 1 ). Repeated screening was recommended for those with suboptimal image quality. For interpretation, DICOM images were uploaded to a proprietary cloud computing environment (SigTel®, UFMG, Belo Horizonte, Minas Gerais, Brazil) for online reporting utilizing commercially available imaging software,^14^in Brazil and the United States (CAS, MCN, CS). All reports were made available online for the PC centers, informing that it was not a final diagnosis.

**Figure 2:** Simplified screening protocol for handheld devices for evaluation of heart diseases in adults, consisting of 14 images (7 views): 10 loops and 4 still frames. AP4: apical four chambers; AP5: apical five chambers; AO: aorta; AV: aortic valve; AR: aortic regurgitation; LA: left atrium; LV: left ventricle; MR: mitral regurgitation; MV: mitral valve; PLAX: parasternal long axis; PSAX: parasternal short axis; PV: pulmonary valve; RA: right atrium; RV: right ventricle; TV: tricuspid valve.

### Statistical analysis

Data were entered to the proprietary SigTel® cloud and exported to the RedCap® database.^15^Statistical analysis was performed using SPSS® software version 23.0 for Mac OSX (SPSS Inc., Chicago, Illinois, United States). As an exploratory study, no pre-specified sample size calculation was performed, and all eligible individuals undergoing tele-ECG screening in 17 months were enrolled. The Shapiro-Wilk test was used to assess the distribution of continuous variables. Categorical variables, expressed as numbers and percentages, were compared between groups (with and without major HD in echo screening) using Fisher's exact test, whereas continuous data, expressed as median and Q1/Q3 (25%/75%), were compared using the Mann-Whitney U test, as appropriate.

Multivariable logistic regression was used to examine the association between the presence of major ECG abnormalities according to the Minnesota code and the presence of major HD in screening echo. Significant variables (p < 0.10) in univariate analyses were included in multivariate models. Four regression models were adjusted as follows: 1) unadjusted model; model 2) model 1 adjusted for age and sex; model 3) model 2 plus cardiovascular risk factors (hypertension and diabetes); model 4) model 3 plus history of cardiovascular disease (Chagas, rheumatic heart disease [RHD], ischemic heart disease, stroke, and heart failure). All clinical variables considered were collected during the clinical interview. For all analyses, a two-tailed significance level of 0.05 was considered statistically significant.

## Results

After the initial training period, the quality of the acquired exams was graded as satisfactory for interpretation in > 90% of the 50 initial cases during the quality assurance period, and patient enrollment was then initiated.

A total of 1,411 patients underwent screening echo in 17 months. Based on the Minnesota code, 1,149 (81%) had major ECG abnormalities, and 19% were enrolled in the control group. Median age was 67 (IQR 60 to 74) years; 51.4% were male; 76.3% had hypertension, 37.7% diabetes, and 22.3% ischemic heart disease. Although the rates of preexisting heart failure, Chagas disease and RHD were overall low, the clinical and demographic variables depict a high-risk profile of the sample. Overall, patients found to have major HD in echo screening were older, and they had higher prevalence of hypertension and diabetes, as well as higher rates of previously known heart failure and ischemic heart disease ( Table 1 ).

**Table 1:** Characteristics of the included sample and comparison of demographic and clinical variables between patients with and without major heart disease detected by screening echocardiography

Characteristic		N	Overall^1^	Major echo abnormality (n=475)	Normal (n=936)	p value^2^
Sex		1,411				0,092
	Female		686 (48.6%)	216 (45.5%)	470 (50.2%)	
	Male		725 (51.4%)	259 (54.5%)	466 (49.8%)	
Age (years)		1,411	67.0 (59.5; 74.0)	73.0 (64.0; 80.0)	64.0 (58.0; 71.0)	**<0.001**
ECG		1,411				**<0.001**
	Major abnormality		1,149 (81.4%)	424 (89.3%)	725 (77.5%)	
	Normal		262 (18.6%)	51 (10.7%)	211 (22.5%)	
Follow-up echo promptly recommended		1,411	368 (26.1%)	334 (70.3%)	34 (3.6%)	**<0.001**
Hypertension		1,411	1,076 (76.3%)	397 (83.6%)	679 (72.5%)	**<0.001**
Diabetes		1,411	532 (37.7%)	197 (41.5%)	335 (35.8%)	**0.037**
Chagas disease		1,411	13 (0.9%)	6 (1.3%)	7 (0.7%)	0.381
Heart failure		1,411	38 (2.7%)	27 (5.7%)	11 (1.2%)	**<0.001**
AMI/coronary artery disease		1,411	314 (22.3%)	142 (29.9%)	172 (18.4%)	**<0.001**
Rheumatic heart disease		1,411	4 (0.3%)	1 (0.2%)	3 (0.3%)	>0.999
Stroke		1,411	78 (5.5%)	32 (6.7%)	46 (4.9%)	0.157

1 n (%); median (interquartile range).

2 Pearson's chi-squared test; Wilcoxon rank sum test; Fisher's exact test. AMI: acute myocardial infarction; ECG: electrocardiogram; echo: echocardiogram.

A total of 475 (33.7%) patients had major HD in screening echo. Among those with major ECG abnormalities, 37% (N = 424) had abnormal echo, compared to 19% (N = 51) of those without major ECG findings (p < 0.001). After consensus evaluation of the screening images, a priority follow-up echo was recommended for 334 patients (70.3%) with major HD in screening, and for 34 (3.6%) of those without abnormalities, for diagnostic clarification or due to suboptimal images.

In regression models, major ECG abnormalities by the Minnesota code were associated with a 2.4 higher chance of having major HD in screening echo in the bivariate analysis, and remained significant (p < 0.001) after adjustments in model 2 (OR = 2.57), model 3 (OR = 2.52), and model 4 (OR = 2.23) ( Table 2 ). Despite multiple adjustments, the association between ECG and echo abnormalities remained strong in model 4, with OR > 2.0 and p value < 0.001 ( Table 2 ).

**Table 2:** Adjusted regression models (4) to assess the association between major tele-ECG abnormalities by the Minnesota Code and the presence of major heart disease detected by screening echocardiography

Variables/model		OR (95% CI)	p value
**Model 1**			
	(Intercept)	0.24 (0.18-0.33)	**< 0.001**
	Major ECG abnormality	2.42 (1.76-3.39)	**< 0.001**
**Model 2**			
	(Intercept)	0 (0-0)	**< 0.001**
	Major ECG abnormality	2.57 (1.84-3.65)	**< 0.001**
	Sex	1.37 (1.08-1.74)	**0.009**
	Age	1.07 (1.06-1.08)	**< 0.001**
**Model 3**			
	(Intercept)	0 (0-0)	**< 0.001**
	Major ECG abnormality	2.52 (1.80-3.58)	**< 0.001**
	Sex	1.41 (1.11-1.79)	**0.005**
	Age	1.07 (1.06-1.08)	**< 0.001**
	Hypertension	1.46 (1.07-2.00)	**0.017**
	Diabetes	1.05 (0.82-1.35)	0.688
**Model 4**			
	(Intercept)	0 (0-0)	**< 0.001**
	Major ECG abnormality	2.23 (1.59-3.19)	**< 0.001**
	Sex	1.35 (1.06-1.73)	**0.015**
	Age	1.07 (1.06-1.09)	**< 0.001**
	Hypertension	1.32 (0.97-1.81)	0.083
	Diabetes	1.03 (0.80-1.33)	0.825
	Chagas disease	1.09 (0.29-3.82)	0.897
	Heart failure	5.18 (2.43-11.87)	**< 0.001**
	AMI/coronary artery disease	1.56 (1.18-2.07)	**0.002**
	Rheumatic heart disease (known)	0.37 (0.01-6.16)	0.491
	Stroke	1.18 (0.72-1.93)	0.504

AMI: acute myocardial infarction; ECG: electrocardiography.

Among the demographic and clinical variables considered for the models, age (each 1 year), male sex, and previous history of heart failure and acute myocardial infarction/coronary artery disease were independent predictors of major HD on screening echo, in addition to major ECG abnormalities ( Table 2 ).

## Discussion

Our proof-of-concept study showed that a combination of tele-cardiology tools may be a promising strategy for screening for HD and prioritization of referral tests in the PC setting. Major abnormalities in the tele-ECG (a widespread telehealth resource in Brazil) adequately predicts the presence of major HD in echo screening with remote interpretation, independently of clinical variables. These findings point towards the utility of such tools to improve cardiovascular care in low-resourced regions, through the potential incorporation of low-cost and available modalities into clinical risk scores. In Brazil, access to health care is unequal,^16^and mortality rates associated with cardiovascular disease also vary depending on regional characteristics and socioeconomic conditions.^1^Therefore, decentralization of health services is a challenge and a growing necessity in the country in order to provide better access to healthcare for the population in need.

Accordingly, in addition to improving efficiency and reducing costs, telemedicine can expand the boundaries of PC, providing access to healthcare for remote populations and potentially expanding the actions of health professionals, integrating them with specialized health services located in hospitals and reference centers and ultimately democratizing access to prevention, diagnosis, and treatment.^8 , 17^However, it is known that Brazil has numerous structural drawbacks for achieving universal access to telehealth devices, especially related to adequate access to internet connection. This situation tends to be even worse in remote and peripheral regions, representing a significant barrier for the dissemination and consolidation of telemedicine in the country.^1 , 2^Even if the technical infrastructure for telemedicine is solved in Brazil, there will still be a risk of shortage of local capacity and personnel to promote the expansion of access to healthcare, given that remote services are essentially inter- and multidisciplinary.^2^Thus, consolidation of partnerships between public and private sectors is an essential step to make telehealth resources available and effectively functional in multiple locations, given the country's continental dimensions.

Aimed at addressing these issues, government programs have implemented tele-ECG as one of the priority telehealth tools in several regions of the country in the past decades,^8 , 18^taking advantage of partnerships between the PC of several Brazilian municipalities and university hospitals, where the ECG tracing is interpreted in real-time, and reports are immediately sent back to the basic units.^8^Tele-ECG has proven to be a robust telehealth tool for locations far from large urban centers with limited access to specialized health services.^8 , 9^Available data consolidated it as a valid and cost-effective tool for improving diagnostic and therapeutic accuracy in places where lacking specialists, in addition to promoting healthcare for populations with suboptimal access.^9 , 19^Our data reinforce the diagnostic utility of tele-ECG from a broader perspective, with optimal prediction of major HD detected by screening echo, even after adjustment for relevant clinical variables.

In this scenario, the combination of telecardiology tools for cardiovascular risk stratification in PC appears as a promising strategy, considering the possibility of integration of echo screening performed by non-physicians with remote interpretation by certified cardiologists into usual care.^6 , 20^It is worth highlighting that screening echo with handheld devices, as tested in our protocol, is not proposed as a tool for final diagnosis, with detailed analysis of morpho-functional variables; rather, it is intended for fast acquisition of simplified ultrasound protocols by non-medical staff with brief technical training, aimed at flagging major abnormalities for triage purposes.^21 – 23^Individuals with abnormal findings must ideally undergo a confirmatory standard echo with a comprehensive protocol, focused on deeper assessment of morpho-functional changes.^21^Essentially, simpler and faster screening protocols are easier to replicate by non-medical personnel briefly trained for probe positioning, potentially facilitating the deployment of the strategy to more regions, integrated with PC.^6 , 24^In Brazil, however, this task-shifting approach is only allowed in research protocols, as ultrasound images can be acquired exclusively by certified physicians. Discussions about such regulations with policymakers and medical councils are warranted to allow for the expansion of the strategy, if proven feasible and cost-effective, beyond research.

Studies focused on RHD have demonstrated that the screening strategy is effective for early diagnosis,^22 , 23^and it admittedly improves prognosis.^25^Screening echo is usually more sensitive than specific for detection of latent RHD.^21 , 24^For other cardiac pathologies, screening findings have a significant correlation with standard echo performed by specialists.^21^Even though there are recognized reasons for the discrepancies between findings of screening and standard echo,^10 , 21^the experience and training background of scanners, in addition to the limited features of the ultraportable screening devices compared to the fully functional standard machines, are additional factors that account for disagreement. Thus, investments in staff education, training, and quality assurance and collaborative technological development are key approaches for better results.^21^

Considering this, the association between tele-ECG findings and major HD detected by screening echo with remote reading raise the possibility of combining such tools in the future, optimizing the utilization of health resources, avoiding transportation costs, and rationalizing referrals from primary to secondary care levels. Our results suggest that the association between major abnormalities in tele-ECG and tele-echo is independent of the patients’ demographic and clinical profiles, as denoted by the multiple adjusted models. This reinforces the hypothesis that a possible combination of these tests would enable more accurate risk stratification in PC.^5 , 7^Furthermore, the possibility of having the patient pre-screened at the primary health center whenever echo is ordered by the attending physician, through a clinical questionnaire, triage tele-ECG and, when indicated, screening echo, would be a feasible way to accurately predict the presence of significant HD, rationalize indications of standard echo and avoid long and sometimes unnecessary referral delays.^26^The feasibility of integrating screening echo into PC has been previously demonstrated by our group, as well as the potential to provide early diagnosis of HD for adults and the elderly.^5 , 6^

The scenario of Brazilian public secondary care is challenging in most states, with long delays for complementary cardiovascular exams and appointments, especially in areas with difficult access, small municipalities, and regions far from large urban centers.^1 , 2^Long waiting times frequently represent a delay for initiation and optimization of treatment. In this context, a more precise risk stratification strategy might be useful both to adapt patient's treatment and to reduce the demand for referrals, ultimately leading to shorter waiting lists. Based on our findings, the possibility of pre-triage with tele-ECG is already a reality, with compelling initial results. Additionally, the possibility of having semi-automated ECG diagnosis, as already available in the Telehealth Network of Minas Gerais,^18^and machine learning algorithms for ECG^27^and echo^28 , 29^may further boost the predictive power of the methods and reduce operational costs.

One question that remains unexplored is the possibility of developing a prediction score combining simple clinical variables, tele-ECG, and screening echo with remote reading. Although the addition of screening echo to clinical variables admittedly improves the predictive power of an existing risk score,^5^the effect of including tele-ECG in the model and the prognostic impact of the strategy are still unknown. More studies are warranted to define optimal echo screening imaging protocols (e.g. simplified versus single-view), as well as to establish indications and ideal screening scenarios for optimization of active case-finding. Finally, discussions with medical councils and health authorities are needed in the light of emerging data on this topic, considering that imaging acquisition by non-physicians is not allowed outside research projects in Brazil and other developing countries, even with exclusive interpretation by cardiologists. Corporatist restrictions may be a barrier for the implementation of cost-saving strategies in the cardiovascular field.

### Limitations

Our study has several limitations. First, this is a proof-of-concept implementation study, in which only the accuracy of tele-ECG was assessed, as opposed to its incorporation into a comprehensive risk score. However, a stepwise assessment of the accuracy of individual tests is a crucial step for the development of predictive scores, as planned for further stages of the PROVAR+ study. Second, the gold standard for defining major HD was screening echo with handheld devices, which may have led to diagnostic limitations. In addition, this generation of GE-VSCAN Extend does not have spectral Doppler capabilities. All interpretation, however, was consensually made by at least 2 experienced cardiologists, with expertise in research projects related to screening. Third, no stratified sampling procedures were performed, and enrollment was consecutively done by convenience based on the tele-ECG system, limiting the extrapolation of findings to the Brazilian population. Also, screening echo data from patients non-eligible for screening, based on the ECG results, are not available for comparison. Finally, sample size limits deeper insights about specific associations between individual ECG abnormalities and functional/morphological echo findings. Despite the aforementioned limitations, to the best of our knowledge, this is the largest study conducted in Latin America aimed at evaluating a combined approach of telecardiology tools in PC, and our data point towards the need for further assessment, as planned for the near future.

## Conclusions

Major tele-ECG abnormalities increased the likelihood of major HD on screening echo, even after adjustments for demographic and clinical variables. The combination of tele-cardiology tools and clinical data may improve risk stratification in PC, and the results point towards the development of risk scores encompassing different modalities for prioritization of care.

## *Supplemental Materials

For additional tables, please click here .

For additional figures, please click here .
